# Patient-derived organoids as a model to study tubo-ovarian carcinoma: a pathologist’s perspective

**DOI:** 10.1186/s13048-025-01766-4

**Published:** 2025-08-20

**Authors:** Catarina Alves-Vale, Beatriz Galvão, Ana Rita Silvestre, José Silva Pereira, Li Bei, João Paulo Fernandes, Paula Borralho, Maria Carmo-Fonseca, Noélia Custódio

**Affiliations:** 1 Department of Pathology, CUF Oncologia, Lisboa, Portugal; 2 Gulbenkian Institute for Molecular Medicine, Edifício Egas Moniz Avenida Professor Egas Moniz, Lisboa, 1649-028 Portugal; 3 Faculdade de Medicina, Universidade de Lisboa, Lisboa, Portugal; 4 Gynaecological Oncology Unit, CUF Oncologia, Lisboa, Portugal

**Keywords:** Patient-derived organoids, Tubo-ovarian carcinoma, Fallopian tube, Pre-clinical models

## Abstract

**Background:**

Tubo-ovarian carcinoma, a leading cause of gynaecological-related mortality, holds substantial biological and clinical heterogeneity. Despite advancements in drug development, predicting therapeutic efficacy remains challenging, partly due to the limited availability of in vitro models that accurately replicate tumour behaviour. We present a concise overview of the intrahospital workflow for establishing patient-derived organoids and analyse the morphological and immunophenotypical features of high-grade serous carcinoma (HGSC), serous borderline tumour (SBT)/low-grade serous carcinoma (LGSC), and normal fallopian tube (FT) organoids.

**Results:**

Samples were collected from patients undergoing surgery or paracentesis. Tissue underwent mechanical and enzymatical digestion. Resulting cell suspensions were resuspended in an extracellular matrix substitute for subsequent culture. Despite the low efficacy in establishing HGSC organoids (*n* = 1/7, 14%; 96 days, 11 passages), we successfully established two organoid lines of SBT/LGSC (*n* = 2/2, 100%; 65 days, 7 passages; 134 days, 16 passages) and normal FT (*n* = 2/2, 100%; 73 days, 10 passages; 58 days, 8 passages). HGSC organoids exhibited limited growth and mostly irregular structures, while preserving the p53 immunostaining pattern of the original tumour. SBT/LGSC and FT organoids maintained features of architectural complexity and faithfully recapitulated the original immunoprofile.

**Conclusions:**

This study highlights the need for a multidisciplinary collaboration in both clinical and research settings to establish patient-derived organoids. It emphasises the pivotal contribution of pathologists in meticulous sampling and organoid characterisation. The integration of diverse expertise is essential for maximising the potential of organoids as preclinical tools, advancing our understanding of tubo-ovarian carcinoma, and ultimately improving patient outcomes.

**Supplementary Information:**

The online version contains supplementary material available at 10.1186/s13048-025-01766-4.

## Background

Tubo-ovarian carcinoma is the second leading cause of mortality among gynaecological malignancies, following cervical cancer [1]. It encompasses five main types: high-grade serous carcinoma (HGSC), low-grade serous carcinoma (LGSC), endometrioid carcinoma, clear cell carcinoma and mucinous carcinoma [2–4]. The paradigm has shifted from considering serous carcinoma as a singular type to recognising HGSC and LGSC as distinct entities, with contrasting morphological, immunophenotypical, molecular, and clinical features [3, 5–7]. HGSC, the most frequent histological type, is primarily thought to originate in the fallopian tube (FT) epithelium from the precursor serous tubal intraepithelial carcinoma (STIC) [8–12]. Given its insidious progression and aggressiveness, approximately 75% of patients are diagnosed at advanced stage, frequently accompanied by peritoneal involvement and ascites [2, 13]. In contrast, LGSC, a rare form of ovarian carcinoma, is postulated to arise through a stepwise progression from ovarian epithelial inclusions (with a possible tubal origin), transforming into serous cystadenoma, serous borderline tumour (SBT) and, ultimately, into LGSC [11, 14, 15]. It affects predominantly younger women, follows a more indolent clinical course, but exhibits a poor prognosis in advanced stages, primarily due to its intrinsic relative chemoresistance [11, 16]. The distinct molecular mechanisms underlying LGSC pathogenesis require alternative therapeutic strategies, as platinum-based therapy, the standard for HGSC, often proves less effective for this subtype [11, 14, 15].

Despite significant advances in targeted therapy and biomarker identification, predicting therapeutic sensitivity remains a challenge [6, 17, 18]. One major limitation is the paucity of in vitro models capable of accurately simulating tumour behaviour. Moreover, understanding the early steps of tumorigenesis is vital for developing effective preventive strategies. Recognising this gap, the emergence of organoids – three-dimensional, self-renewing, and self-organising models, recapitulating the properties of the tissue of origin – offers a transformative approach for studying cancer [19, 20]. Organoids can be initiated from pluripotent stem cells or organ-restricted adult stem cells, as in the case of patient-derived organoids [19, 20]. While these models have been established across various types of normal and neoplastic tissue, their implementation presents unique challenges compared to conventional cell culture techniques [19, 21, 22].

In this paper, we present an analysis of the morphological and immunophenotypical properties of patient-derived organoids from HGSC, SBT/LGSC, and normal FT. Despite the small sample size, we aimed to bridge the gap between established pathology protocols in the clinical setting and organoid technology. We discuss key upstream factors that may affect the success of organoid derivation and the challenges encountered within our intrahospital workflow, underscoring the importance of a multidisciplinary collaboration to maximise organoid generation and ensure accurate characterisation of these human in vitro models.

## Materials and methods

Material and reagents references are listed in Supplementary Material.

### Sample collection

**Fig. 1:**
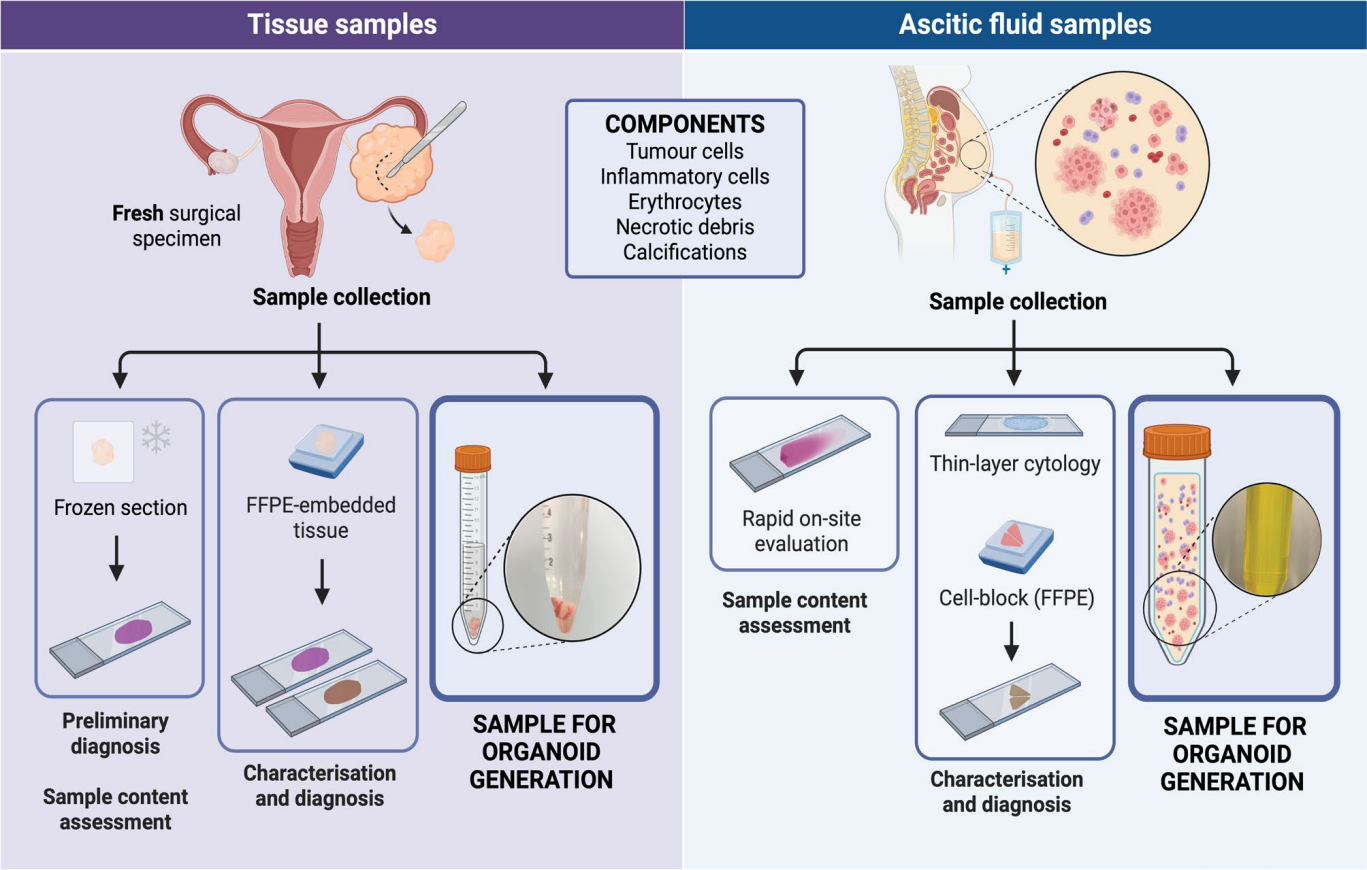
Pathology-guided sample processing for organoid generation. Schematic representation elucidating the sample collection process for patient-derived organoid generation, including assessment of sample quality and histological diagnosis (comprising tissue samples and effusions, e.g. ascitic fluid). Note: Not drawn to scale. FFPE: Formalin-fixed, paraffin-embedded

This study was approved by the Ethics Committee of CUF Descobertas Hospital, Lisbon, Portugal. Eligible patients were identified through the Gynaecological Tumour Board. Samples were collected from consenting patients immediately after surgery or paracentesis (Fig. 1). After pathological assessment through frozen section, cancer tissue samples (5–10 mm-width) were promptly collected, avoiding areas of necrosis, haemorrhage, and hydropic degeneration. Tubal samples were obtained from macroscopically normal salpingectomy specimens of patients undergoing surgery for benign uterine pathology (2 mm-width fragments from the distal part of each tube, excluding fimbria). Samples were transported in cold DPBS and processed within 2–3 h. Ascitic fluid samples were evaluated through a Papanicolaou-stained smear, with a maximum storage time of 3 h at 4 °C before further processing.

### Generation of tubo-ovarian carcinoma organoids

**Fig. 2:**
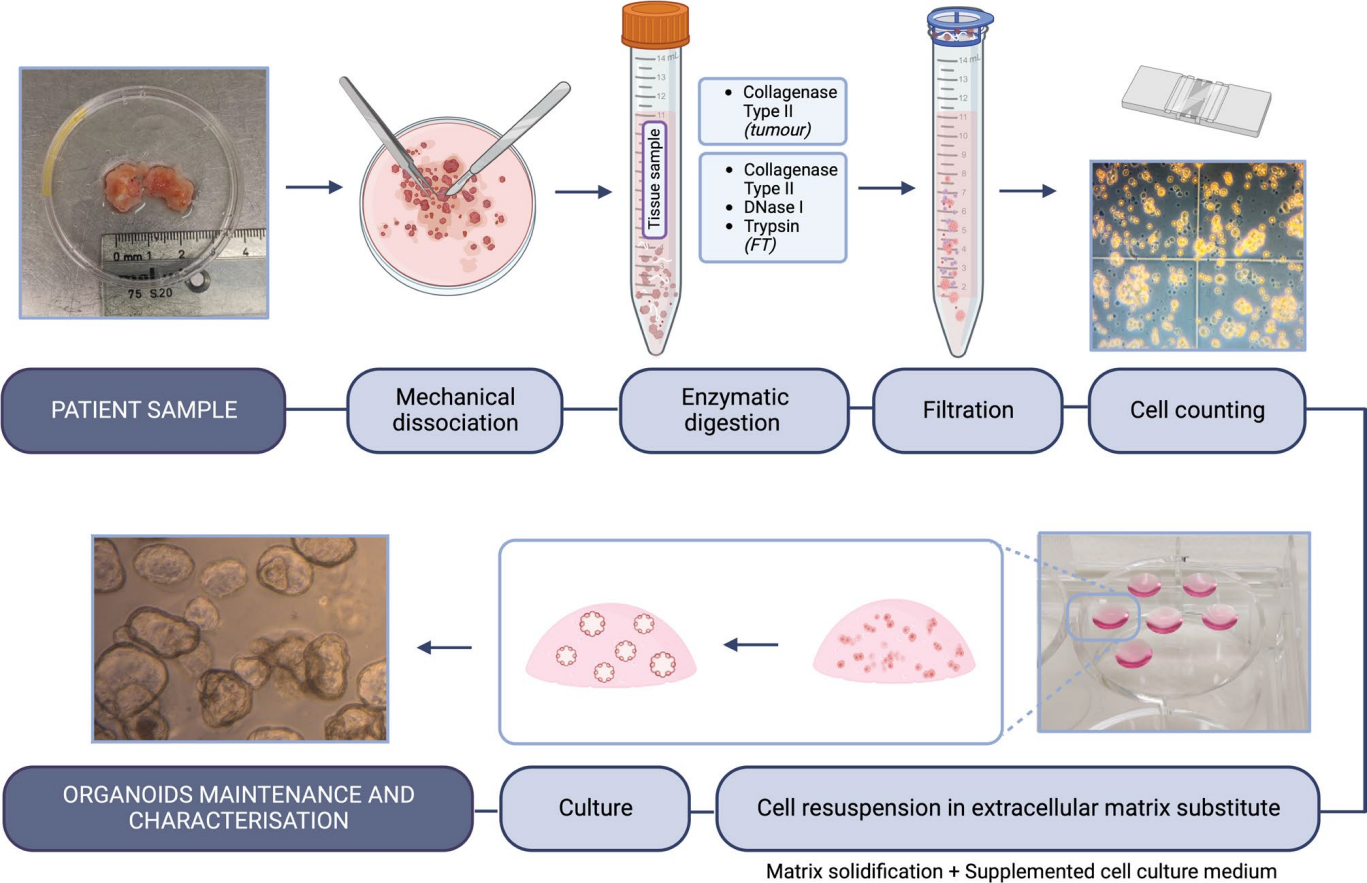
Protocol overview for patient-derived organoid culture. Tissue is processed through mechanical and enzymatic steps, followed by filtration. Cells are resuspended in extracellular matrix substitute and plated in droplets, with culture medium. Organoid cultures are characterised by morphological and immunophenotypical analysis. Note: Not drawn to scale

Tissue samples were diced into 2-mm fragments in basal medium (Advanced DMEM/F-12, 1x GlutaMax, 1% (v/v) HEPES and 1% (v/v) Penicillin-Streptomycin), containing Type II Collagenase (2.5 mg/mL), using sterilised scalpels and tweezers (Fig. 2) [23]. After incubation for 30–45 min at 37 °C with agitation, samples were filtered through a 70 μm strainer, and spun at 300xg for 10 min at 4 °C. When hematic, the resulting pellet was washed with ACK Lysing Buffer followed by washing in basal medium and pelleting as before. For effusions, the digestion steps were skipped. Cell counting was performed using a Neubauer chamber.

For culture, 45 µL-droplets of phenol red-free Matrigel^®^ (basement membrane extract), each containing ~ 30 000–40 000 cells, were placed on a pre-warmed 6-well plate [24]. The plate was left upside down in an incubator (37 °C for 20 min) for solidification of Matrigel^®^. 2 mL of tumour organoid complete medium were then added [Basal medium supplemented with 100 ng/mL Noggin, 100 ng/mL R-Spondin1, 1x B27, 1.25 mM N-Acetyl-L-Cysteine, 10 mM Nicotinamide, 5 µM A83-01, 100 ng/mL FGF-10, 100 ng/mL EGF, 10 ng/mL FGF-2, 1 µM Prostaglandin E2 and 10 µM SB 202190] [23]. 10 µM ROCK inhibitor Y-27632 was added upon initial plating, but removed at first medium change. Medium was changed every 2–3 days.

### Generation of normal fallopian tube organoids

FT samples were cut into 2-mm fragments in basal medium (same as for tumour), with 0.5 mg/mL DNase I, 2 mg/mL trypsin from bovine pancreas and 0.5 mg/mL Type II Collagenase (Fig. 2). This mix was incubated for 45 min at 37 °C with agitation. The subsequent steps were as previously described for tumour organoids, with the following modifications on medium: addition of 25% (v/v) Wnt-3 A conditioned medium, change of R-Spondin1 concentration (500 ng/mL), and absence of FGF-2, Prostaglandin E2, and SB 202190 [23].

### Organoids subculture

Organoids were passaged at suitable ratios (1:1–1:3, depending on confluency). After incubation with pre-warmed (37 °C) Accutase^®^ solution, for up to 5 min at room temperature, organoids were mechanically sheared through a P1000 pipette tip, transferred to a Falcon^®^ tube with basal medium for centrifugation (300xg for 10 min at 4 °C), resuspended in basal medium and mixed with Matrigel^®^ for reseeding as above. Cultures were observed on a Zeiss Primovert bright-field inverted microscope (Zeiss AxioCam Erc5s camera) every 2–3 days.

### Histological and immunohistochemical study

Medium was removed, wells were washed with 1x PBS and buffered formalin (10%) was added. Organoids were then transferred to a 15 mL tube with a Pasteur pipette and later resuspended in warm HistoGel™, solidified at 4 °C for 5–10 min, and then processed and paraffin-embedded. 3 μm sections on positively charged slides were stained with H&E and, after ULTRA CC1 antigen retrieval, processed on Ventana BenchMark ULTRA automated staining platform with pre-diluted anti-PAX-8, anti-WT1, anti-p53, anti-ERα, anti-PR, and/or anti-Ki67. Antibodies were run using ultraView DAB detection kit (for anti-ERα and anti-PR) or OptiView DAB IHC Detection Kit (for the remaining antibodies). All slides included an external immunohistochemical tissue control to ensure staining accuracy.

### Immunofluorescence of whole-mount organoids

Upon organoid passaging, 10 µL-Matrigel^®^ droplets were plated in a µ-Slide 8 Well chambered coverslip and covered with complete medium. Once ready for fixation, medium was removed, the wells were rinsed with 1x PBS and organoids were fixed with gentle swirling with 4% (w/v) PFA in 1x PBS for 20 min, at room temperature. After washing with 1x PBS (3 × 10 min each), organoids were stored at 4 °C. For permeabilisation, organoids were incubated with 0.5% (w/v) Triton™ X-100 in 1x PBS for 20 min at room temperature with gentle agitation. Blocking was performed using 3% (w/v) BSA in 1x PBS, for 45 min at room temperature. Rabbit anti-Ki-67 (1/100) and mouse anti-EpCAM conjugated with FITC at 5 µg/mL (2.5/100), diluted in 1.5% (w/v) BSA in 1x PBS, were used as primary antibodies. After overnight incubation, in a humid chamber at 4 °C, each well was washed with 1x PBS containing 0.5% Tween^®^ 20 (3 × 10 min each). The secondary antibodies used were horse anti-mouse IgG DyLight^®^ 488 (1/200) and goat anti-rabbit IgG DyLight^®^ 594 (1/200) diluted in 1.5% (w/v) BSA in 1x PBS. Organoids were incubated with 150 µL of the respective antibody, in the dark, for 1 h at room temperature. Subsequently, were rinsed twice and washed with 1x PBS containing 0.5% Tween^®^ 20, followed by a 10 min incubation with 0.5 µg/mL DAPI in 1x PBS and washed in 1x PBS containing 0.5% Tween^®^ 20 (3 × 5 min each). After the final wash, 100 µL of mounting medium made in-house [9.2 mM p-Phenylenediamine in 90% (w/v) sterile glycerol and 1x PBS, pH 9.5] was added to each well. Images were acquired on a Zeiss LSM 710 confocal point-scanning microscope (Plan-Apochromat 20x/0.8 M27 objective).

## Results

### Successful generation of patient-derived tubo-ovarian carcinoma and normal fallopian tube organoids

Tubo-ovarian carcinoma samples and normal (unmatched) fallopian tube (FT) tissue were obtained from consenting patients undergoing adnexal tumour resection and/or salpingo-oophorectomy for benign pathology, respectively. Additionally, ascitic fluid was collected from patients submitted to paracentesis for confirmed or suspected adnexal malignancy. Tumour and normal FT fresh tissue samples were collected after rapid pathological assessment (Fig. 1). Tissue was then mechanically and enzymatically dissociated (Fig. 2). Cell suspensions were filtered, followed by resuspension in an extracellular matrix substitute and plating with supplemented medium (Fig. 2) [23, 24].

We considered that organoids were successfully established if they displayed stable growth for over four passages. Despite the low efficacy in establishing HGSC organoids (*n* = 1/7, 14%; 11 passages), we successfully established organoids from two SBT/LGSC – cultured for 65 days (7 passages) and 134 days (16 passages). We also generated normal FT organoids from two patients– expanded for 73 days (10 passages) and 58 days (8 passages). Table 1 provides clinical information and time in culture for each sample.

**Table 1:** Clinicopathological features of each sample used for organoid derivation and respective culture duration

Sample	Patient age (years)	Stage (FIGO)	Timing of sample collection	CT (response)	BRCA status	Type of sample	Days (Passages) in culture
HGSC_1	53	IIIA1i	At primary surgery	NACT (partial response– CRS2)	*BRCA1* intron 16 rearrangement (S) (G - NA)	Surgical	15 (P0)
HGSC_2	80	IA	At primary surgery	No	NA	Surgical	12 (P0)
HGSC_3	49	IIB	At primary surgery	No	Neg (G + S)	Surgical	96 (P11)
HGSC_4	49	IIIB	At relapse	Under CT	Neg (G + S)	Ascites	30 (P3)
HGSC_5	71	IIIB	At primary surgery	NACT (partial response– CRS2)	*BRCA1* c.4084G > A p.(Asp1362Asn) (S) (G – Neg)	Surgical	7 (P0)
HGSC_6	49	IIC	At primary surgery	No	*BRCA1* c.1969 C > T p.(GIn657*) (G + S)	Surgical	14 (P0)
HGSC_7	86	IVA	At diagnostic paracentesis	No	NA	Ascites	20 (P1)
SBT/LGSC_1	37	IIIA2	At primary surgery	No	Neg (NA)	Surgical	65 (P7)
LGSC_2	39	IIIB	At primary surgery	No	Neg (G + S)	Surgical	134 (P16)
FT_1	48	NA	Surgery for benign uterine pathology	NA	NA	Surgical	73 (P10)
FT_2	85	NA	Surgery for benign uterine pathology	NA	NA	Surgical	58 (P8)

Characterisation of the fallopian tube (FT), serous borderline tumour (SBT)/low-grade serous carcinoma (LGSC) and high-grade serous carcinoma (HGSC) samples, regarding patient age, tumour staging, timing of sample collection, concomitant/prior history of chemotherapy (and response, if applicable), *BRCA* gene mutational status, type of sample and number of days and respective passage before culture discontinuation. Only HGSC cultures were discontinued by having reached a steady-state. FIGO - International Federation of Gynecology and Obstetrics; NA - Not applicable/ available; CRS– Chemotherapy Response Score; CT– chemotherapy; G– Germline mutation; S– Somatic mutation

### High-grade serous carcinoma organoids exhibited limited grow in culture

Samples of HGSC obtained from seven patients, including two ascitic fluids, were processed for culture. Two patients had been submitted to neoadjuvant chemotherapy, and one patient was under chemotherapy at the time of sampling (Table 1). HGSC culture establishment efficacy was lower (*n* = 1/7, 14%) than for SBT/LGSC and FT (100%) (Table 1), presenting less cellularity and a slower cell culture growth rate. Long-term culture was only possible for one sample (HGSC_3) (96 days, 11 passages). Notably, organoids derived from *BRCA1*-mutated tumours tended to exhibit a more limited growth, prompting early culture discontinuation – HGSC_1, HGSC_5 and HGSC_6 (Table 1).

**Fig. 3:**
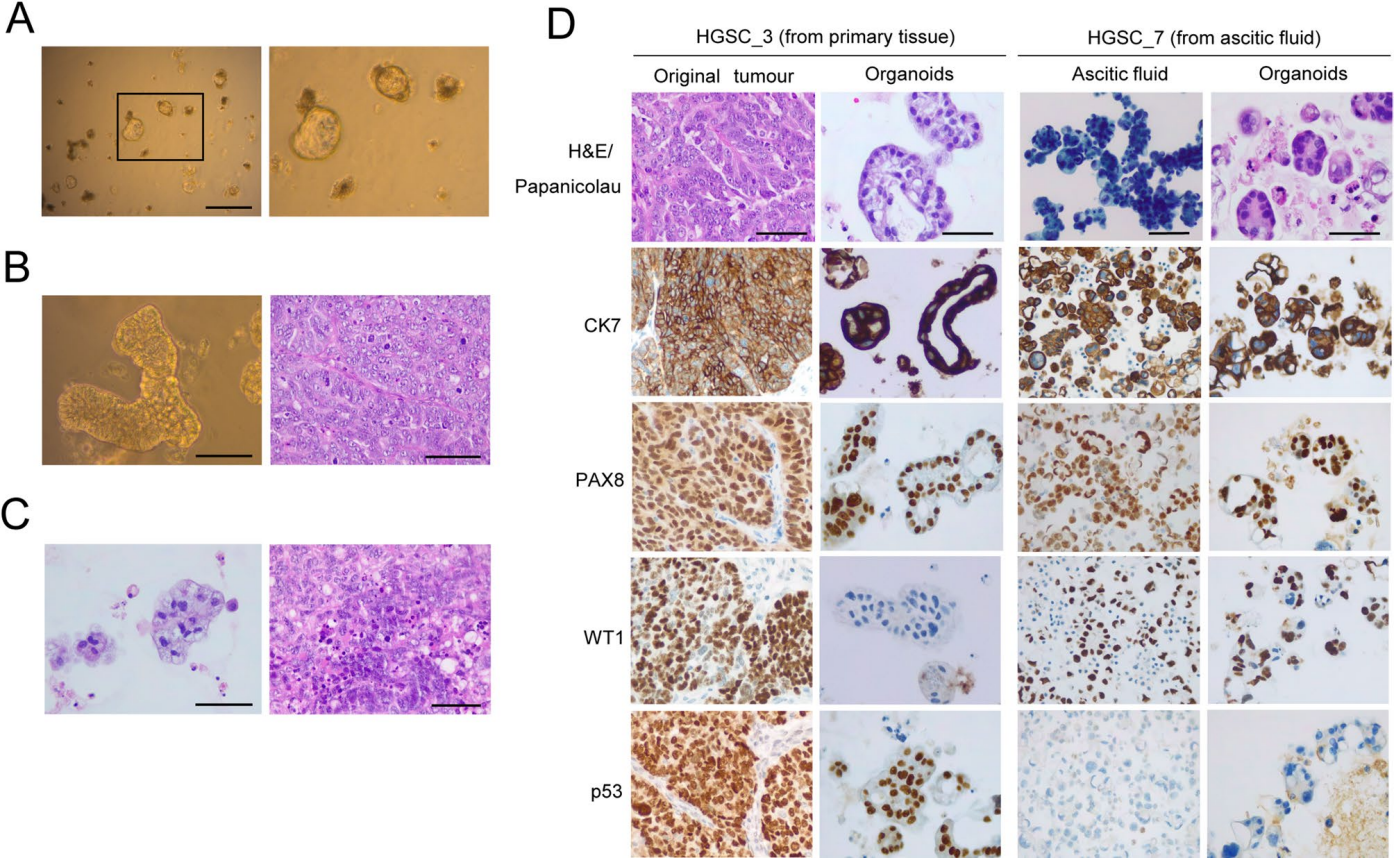
High-grade serous carcinoma (HGSC) organoids exhibited limited growth in culture, but retained p53 expression pattern. A– Representative bright-field images of HGSC organoids (HGSC_3, P11 D11), showing regular spherical structures alongside irregular, discohesive aggregates (zoom in, left). Scale bar: 500 μm. B– Bright-field images of HGSC organoids (HGSC_3, P10 D6) on the left, mimicking the trabecular architecture observed in the primary tumour (right), with peripheral palisading. Scale bar: 100 μm. C– Frequent apoptotic bodies were observed in culture (left), in concordance to the original tumour (right). Scale bar: 100 μm. D– Organoids maintained moderate to marked cytological atypia, while retaining the expression of CK7 and PAX8. WT1 expression was lost in HGSC_3, but retained in HGSC_7 organoids (note: histiocytes, which are WT1-, are also identified in the ascitic fluid). The “mutant-type” pattern of p53 expression is concordant between the original neoplasm and the respective organoids - overexpression pattern in HGSC_3 (expression in >80% of cells), and null pattern in HGSC_7 (absence of staining, with appropriate external controls). Scale bar: 100 μm

HGSC cultures presented mostly irregular discohesive aggregates and, at a lesser degree, regular and organised structures, either cystic or trabecular, sometimes mirroring exactly the original tumour architecture (Fig. 3A, B). Organoids showed marked cytological atypia and frequent apoptotic bodies, which were also observed in the corresponding parental samples (Fig. 3C, D).

Immunohistochemical analysis revealed diffuse positivity for CK7 (cytokeratin 7), a low molecular weight cytokeratin expressed in a variety of epithelial tissues, including tubo-ovarian neoplasms, and for PAX8 (paired box 8), a marker of Müllerian lineage, in both the original tissue and the corresponding organoid lines [25–27]. In contrast, expression of WT1 (Wilms Tumour 1), a characteristic marker of serous neoplasms, was lost in HGSC_3 organoids (Fig. 3D) [27, 28]. Notably, a heterogeneous pattern of staining was observed in the corresponding primary tumour (Fig. 3D). Moreover, over 95% of HGSC cases are described to exhibit *TP53* mutations, and the type of mutation strongly correlates with the staining pattern [27, 29, 30]. Indeed, the analysed organoids showed a “mutant-type” p53 expression pattern, either null or of overexpression, in concordance with the original neoplasm (Fig. 3D) [31, 32]. However, the low cellularity of these cultures precluded further characterisation.

### Long-term organoids derived from serous borderline tumour/low-grade serous carcinoma reflected the architectural complexity and the immunoprofile of the corresponding neoplasms

We successfully generated organoids from two cases of LGSC (Table 1). Of significance, in one case (SBT/LGSC1), the histological examination of the fragment juxtaposed to that utilised for culture (referred to as ‘mirror’ fragment) exhibited SBT morphology, despite a definitive diagnosis of LGSC following extensive analysis of the surgical specimen, highlighting tumour heterogeneity.

**Fig. 4:**
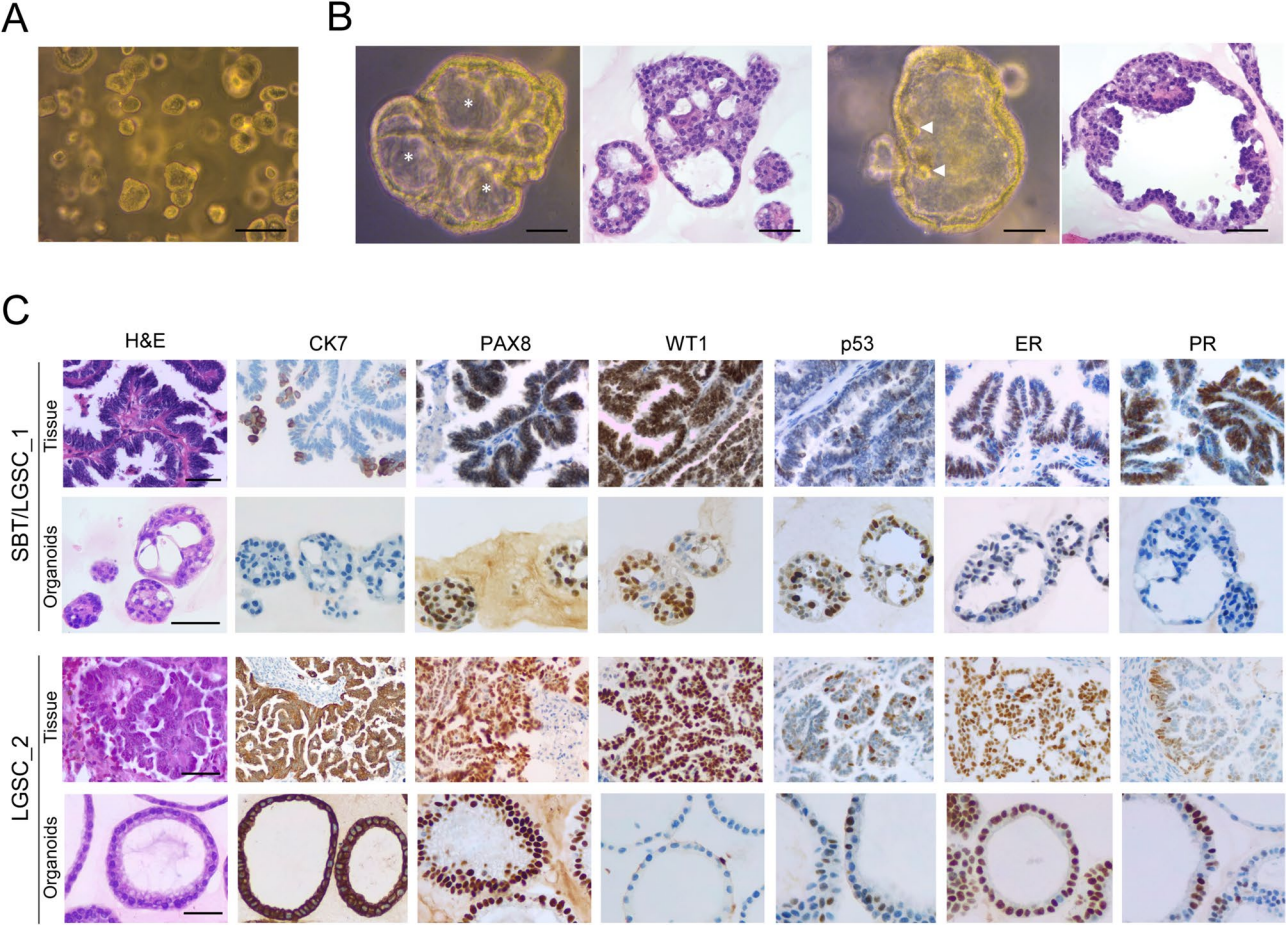
Architectural and immunophenotypical fidelity of serous borderline tumour/low-grade serous carcinoma (SBT/LGSC) organoids. A– Representative bright-field image of early culture (LGSC_2, P0 D7), exhibiting predominantly hollow structures with diverse shapes. Scale bar: 500 μm. B– Bright-field images revealed cribriform (right, * indicating multiple lumens) and micropapillary (left, arrowheads indicating projections) patterns in SBT/LGSC organoids (SBT/LGSC_1, P2 D6), with corresponding histology (SBT/LGSC_1, P7). Scale bar: 100 μm. C– Immunohistochemical study illustrating overall tissue-organoids concordance: CK7 was focal in SBT/LGSC_1 tissue, with absence of staining in organoids, but LGSC_2 tissue and organoids showed a strong and diffuse positivity; PAX8 was also diffusely positive, despite a variable WT1; p53 immunostaining exhibited a wild-type pattern; SBT/LGSC_1 organoids (P2) displayed focal weak positivity for estrogen receptors (ER) and absence of staining for progesterone receptors (PR), while LGSC_2 (P5) accurately mirrored the original tumour. Scale bar: 100 μm

SBT/LGSC organoids exhibited distinct stages of maturation, predominantly with a spherical morphology (Fig. 4A) and occasional cribriform and/or micropapillary architecture, demonstrating their ability to preserve complex features in culture (Figs. 4B and 6A). Organoids were composed of cubic to columnar cells, primarily oriented towards the lumen, with minimal nuclear pleomorphism (Fig. 4B, C).

Immunohistochemistry analysis revealed concordance between the organoids and the parental tumours (Fig. 4C). Both showed positivity for the Müllerian marker PAX8, although inconsistently for CK7 and WT1 (Fig. 4C) [27, 28]. Furthermore, p53 expression in a wild-type pattern aligns with the characteristic absence of *TP53* mutations in LGSC (Fig. 4C) [4, 27, 33]. Considering the potential use of hormonotherapy in LGSC, we performed immunohistochemistry for estrogen receptors (ER) and progesterone receptors (PR) [16]. Notable heterogeneity in expression was observed within the tissue of both tumours (Fig. 4C). In organoids, SBT/LGSC_1 showed a reduction in ER expression with loss of PR expression, while LGSC_2 organoids maintained consistent expression for both receptor types (Fig. 4C).

### Fallopian tube organoids recapitulated mucosal architecture and immunophenotype

**Fig. 5:**
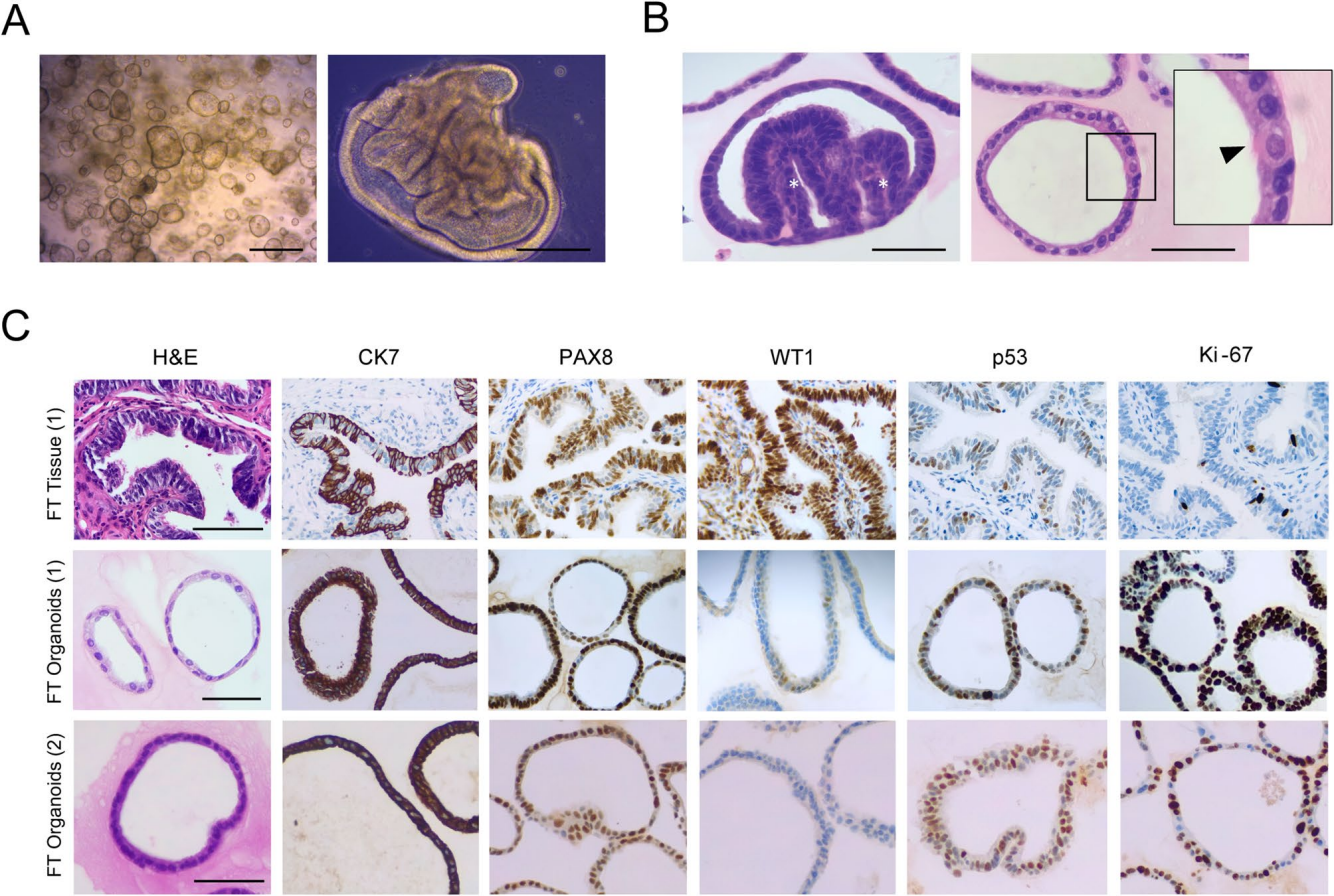
Fallopian tube (FT) organoids captured essential architectural features and the immunophenotype of normal human FT. A– Bright-field image of early FT organoid culture (FT_2, P1 D7), displaying spherical structures (left). Occasional intraluminal projections resembling the development of mucosal plicae were noticed (FT_1, P10, D12) (right).  Scale bar: 500 μm (left), 100 μm (right). B– Histological analysis confirmed the presence of plicae-like epithelial projections (left; FT_1, P2). However, organoids were mainly composed of a simple monolayered, polarised epithelium, featuring secretory and ciliated cells (arrowhead) (right; FT_1, P3). Scale bar: 100 μm. C– FT organoids expressed CK7 and the lineage marker PAX8, with focal staining for WT1. CK7 and PAX8 highlight secretory cells, while ciliated cells are PAX8-negative. Despite a high p53 positivity, a wild-type pattern is maintained, accompanied by an elevated proliferative index (Ki-67). Scale bar: 100 μm

**Fig. 6:**
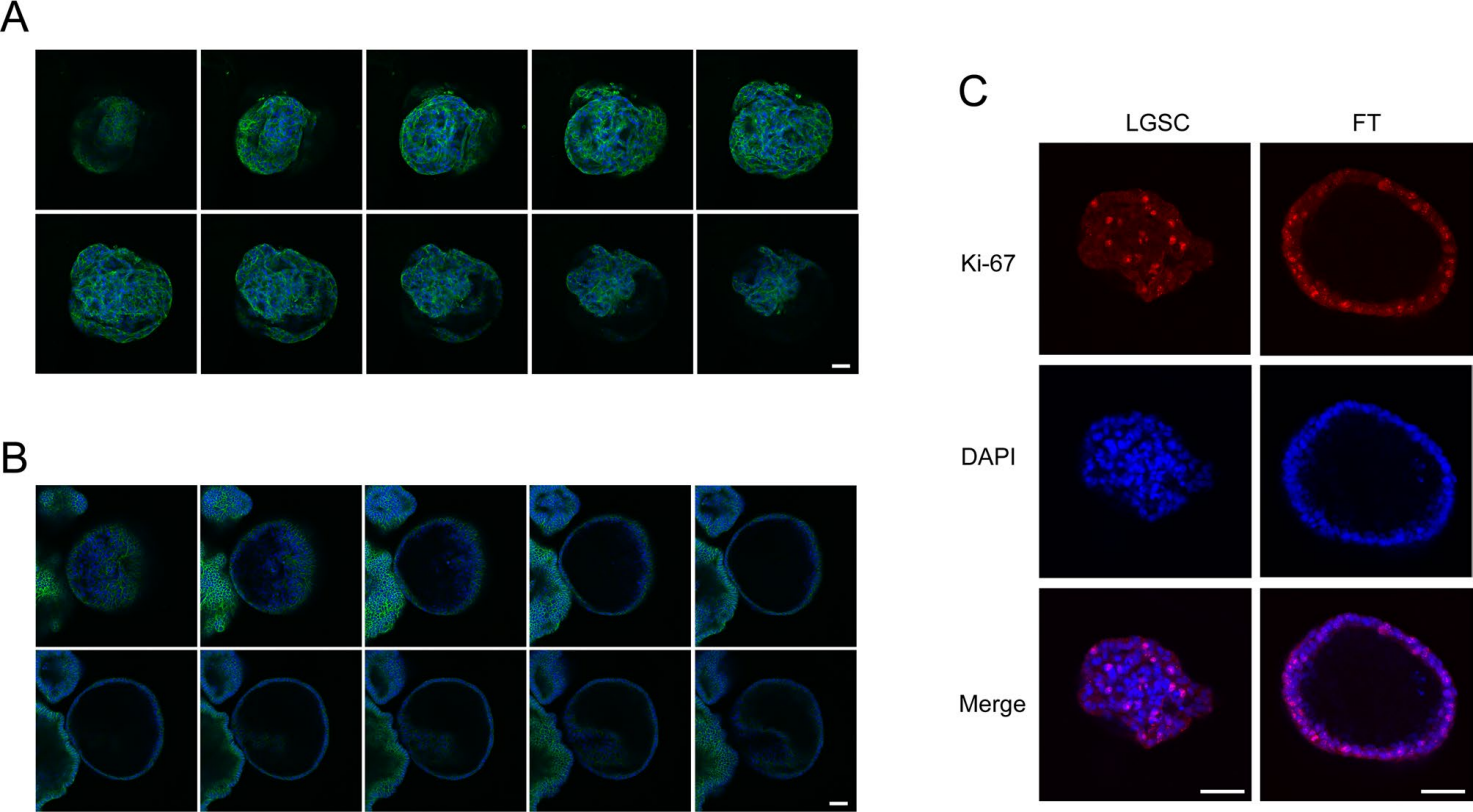
Immunofluorescence of whole-mount patient-derived low-grade serous carcinoma (LGSC) and normal fallopian tube (FT) organoids. A, B  –  Organoids were stained for the epithelial marker EpCAM (green) and nuclear marker DAPI (blue), revealing differences in morphology. LGSC organoids (A) formed denser and more complex structures harbouring multiple lumens, contrasting to the hollow FT organoids (B), with focal invaginations. C– Representative images of organoids stained for the proliferation marker Ki-67 (red) and nuclear marker DAPI (blue) indicating a high proliferative rate in both organoid types. Scale bar: 50 μm

We have successfully cultivated organoids from normal FT epithelium of two patients (Table 1). Organoids were predominantly spherical (Figs. 5A and 6B) and composed of a monolayered epithelium with polarised cubic to columnar cells (Fig. 5B). However, the development of epithelium folds, a feature of architecture complexity, mirroring normal tubal mucosa, was observed in several organoids during culture, and later confirmed by histological analysis (Fig. 5A, B). The presence of both secretory and ciliated cells was morphologically evident, with secretory cells being the prevailing cell type (Fig. 5B, C). FT organoids showed expression of PAX8, a Müllerian marker specifically expressed by the tubal secretory cells, but not in the ciliated population (Fig. 5C) [34, 35]. Organoids were also diffusely positive for CK7, a marker for the secretory cells, highlighting the predominance of this cell type in culture (Fig. 5C) [36].

In contrast, organoid immunoreactivity for WT1, a lineage marker also expressed in the normal FT epithelium, was only focal (Fig. 5C). As anticipated for a normal tissue, organoids displayed high, but wild-type expression pattern for p53 (Fig. 5C). Recognising its function as a checkpoint regulator induced during cell division, we conducted a Ki-67 analysis. Notably, FT organoids were more proliferative than the original tissue (75–90% versus < 5%, respectively) (Fig. 5C), potentially leading to increased p53 expression. Histological examination of FT specimens revealed no evidence of neoplasm. Both FT and LGSC organoids displayed a high proportion of Ki-67 + cells (Fig. 6C), compatible with the high proliferating rates observed in culture.

## Discussion

Tubo-ovarian carcinoma remains a major oncological challenge, with limited improvement on overall survival over the years [13]. In this context, patient-derived cancer organoids have emerged as a promising in vitro model for precision oncology with potential for identification of relevant biomarkers and enabling high-throughput drug screening [19, 37]. Several studies have reported the generation of patient-derived organoids from various types of tubo-ovarian carcinoma, as well as normal tissues comprising both tubal and ovarian surface epithelium [38–41].

HGSC accounts for the majority of cases and is characterised by high genomic instability, ubiquitous presence of *TP53* mutations and, less frequently, mutations in *BRCA1/2* genes, increasing its susceptibility to cellular stress, potentially impairing cell survival and adaptability to ex vivo culture conditions [29, 30, 42]. Successful establishment of organoid cultures has been reported from both chemotherapy-naïve and previously treated HGSC samples. Theoretically, prior exposure to chemotherapy may negatively affect culture establishment, as cytotoxic treatment can induce cellular senescence in a subset of tumour cells [43]. For example, Maenhoudt et al. observed a slightly higher, although non-statistically significant, organoid derivation success rate in chemotherapy-naïve samples compared to those from previously treated patients (56% vs. 36%) [44]. In contrast, Hill et al. reported comparable establishment rates between the two groups, provided that macroscopically visible tumour was present at debulking surgery [23]. However, the success rate was lower in cases exhibiting an “extreme response” to chemotherapy, which we interpret as a Chemotherapy Response Score (CRS) of 3 [45]. Furthermore, their findings suggested that the percentage of viable tumour cells was an additional determinant of successful organoid culture [23]. However, studies in other cancer types have not demonstrated an association between organoid derivation efficiency and tumour cell viability or necrosis, underscoring the multifactorial and context-dependent nature of culture success [46, 47]. In our HGSC cohort (*n* = 7), organoid cultures were derived using specimens from patients aged 49–86 years, encompassing FIGO stages IA–IVA, and included both primary surgical specimens and ascitic fluid (Table 1). Despite initiating cultures from all seven cases, only three (HGSC_3, HGSC_4, HGSC_7) could be propagated beyond passage 0, with only one maintained for 11 passages over a 96-day period. HGSC_1 and HGSC_5 originated from patients with a CRS2 (Table 1), characterised by an “appreciable tumor response amidst viable tumor, both readily identifiable and tumor regularly distributed”. Of note, our samples were obtained from adnexal masses rather than the omentum, the latter being the standard site for CRS assessment [45]. Reported success rates in establishing long-term HGSC organoid cultures range from 23 to 83%, with the latest publications yielding the most favourable outcomes [40, 44, 48–51]. Interestingly, although some authors have reported that Wnt-3 A and R-Spondin1 may limit HGSC organoid growth, the highest efficiency was achieved using a culture medium containing these factors, as originally formulated for FT organoids [48–51]. Additionally, supplementation with β-estradiol has shown to improve HGSC organoid generation, despite not having been used by Hill et al. [23, 51]. In summary, considering that only one of our HGSC samples yielded a long-term culture, the extent to which our findings can be extrapolated is inherently limited. Further validation in larger, independent cohorts is required to validate and expand these observations.

Given the frequently advanced stage of HGSC at diagnosis, it is mandatory to understand the molecular mechanisms underlying malignant transformation, in order to find effective screening and prophylactic strategies. Most cases of HGSC are considered to originate in the tubal fimbria from a precursor lesion - serous tubal intraepithelial carcinoma (STIC) [8–11]. Critical insights into its pathogenesis have predominantly relied on formalin-fixed, paraffin-embedded samples or commercial cell lines, notwithstanding the inherent limitations of these models [52–54]. Patient-derived FT epithelium organoids may constitute a suitable complement for this end [23, 49].

We successfully achieved long-term cultures of FT and SBT/LGSC organoids, which captured the architectural complexity and immunoprofile of the original tissue, thus supporting the reliability of this model (Figs. 4, 5 and 6). Acknowledging that immunohistochemistry offers a very restricted evaluation of gene expression profile, the absence of detailed molecular characterisation constitutes a limitation of this study. An intriguing observation was the partial or complete loss of WT1 immunostaining in some organoids (Figs. 3D, 4C and 5C). This may result from intratumoral heterogeneity – demonstrated, for example, by the variable expression of WT1 in HGSC_3, and also CK7 in SBT/LGSC_1 (Figs. 3D and 4C) – coupled with selection pressure within the culture, induced by media composition, thus favouring specific tumour subpopulations. Indeed, WT1 is a transcription factor pivotal in organogenesis and has been described to stabilise both p53 and β-catenin proteins [55–57]. This interaction led us to hypothesise that WT1 levels might be modulated by the Wnt/β-catenin pathway, through R-Spondin1 and/or Wnt-3 A included in the medium [55, 58]. However, existing evidence suggests that β-catenin safeguards WT1 from degradation, rather than the opposite [55]. While we acknowledge that these mechanisms remain hypothetical without direct experimental validation, they provide a possible framework for understanding WT1 expression changes in our organoid models.

Establishing high-quality, disease-specific biobank collections is essential for advancing organoid-based research, yet it remains challenging [59, 60]. The timely collection and processing of fresh tissue samples involves inherent logistical complexity, necessitating close collaboration among surgical teams, oncologists, pathologists, and scientists.

Various factors associated with sample collection can profoundly impact the viability of organoids and downstream applications. Identification of eligible patients in advance is a critical first step for both effective sample collection and subsequent processing, especially in the setting of a multi-institutional project. Informed consent from all patients should be obtained prior to sample collection. Study specimens may be collected not only during scheduled procedures such as surgeries, but also during urgent clinical episodes (e.g. effusion drainage), underscoring the necessity for effective team-wide communication to maximise the number of eligible samples. The time between tissue excision or effusion drainage and processing must be minimised, as prolonged ischemia induces hypoxic stress, alters gene expression, and compromises cellular viability [61]. Notably, organoids derived from post-mortem samples have also been described [62]. Given that sample collection for organoid derivation is integrated in the standard clinical care, the sampling process must be carefully incorporated into the institutional workflows for routine pathological examination. Accordingly, fresh material can be collected either in the operating room or in the pathology department. Even though antibiotics are often added to culture media, this process should be performed within a laminar flow cabinet using sterilised material.

When obtaining tissue samples for cancer organoid derivation, a careful sampling approach is essential to prevent contamination from normal tissue. Conversely, if the aim is to establish organoids from normal tissue, similar diligence is required to exclude any tumour cells. Also, an initial morphological assessment (e.g., cellularity, necrosis and fibrosis degree), especially after neoadjuvant chemotherapy, can help guide sample selection and subsequent processing, highlighting the pivotal role of pathologists in this procedure (Fig. 1). Both frozen section and rapid on-site cytological evaluation (ROSE) using Diff-Quik or Papanicolaou staining can be used. Cytological samples, either effusions or those obtained through fine-needle aspiration, also proved suitable for organoid derivation, offering an alternative when solid tissue samples are unavailable [23, 49, 63]. Effusion samples contain varying proportions of tumour cells, reactive mesothelial cells, lymphocytes, and macrophages. A ROSE evaluation can help assess tumour cell proportion and determine sample suitability for organoid culture. However, enrichment strategies, such as fluorescence-activated cell sorting (FACS), can be used in both effusions and tissue samples after dissociation to select the cells of interest for further culture [64]. Interestingly, co-culture techniques with stromal cells, such as fibroblasts, endothelial cells or immune cells, have been applied to organoid technology, increasing its complexity [65].

A multi-sampling approach is recommended to account for intratumoral cell heterogeneity, while a longitudinal sampling from the same patient over time (when applicable) can provide insights into tumour evolution and treatment resistance mechanisms [23, 43, 57, 60]. Notably, tubo-ovarian tumours, frequently resected at large sizes, usually offer broader sampling scopes compared to smaller-sized malignancies. In contrast, normal FT sampling should be more cautious. The College of American Pathologists advocates for the total inclusion of the specimen following the Sectioning and Extensively Examining the FIMbriated End (SEE-FIM) protocol in cases of risk-reducing salpingectomies for patients with *BRCA* germline mutations or suspected hereditary risk [45]. Recent European consensus recommendations support using SEE-FIM for any salpingectomy specimen [66]. However, an international panel of expert gynaecopathologists did not reach an agreement based on the low percentage of incidental STIC in the general population and ongoing debate about its clinical impact. Nonetheless, full embedding of the fimbriated portion was recommended [67]. Therefore, when FT tissue is collected for research, adjacent “mirror” fragments should be collected and processed separately for histopathological analysis. Additional serial sectioning can improve STIC detection [67]. Proper annotation of the anatomical regions from which FT fragments are collected (isthmus, ampulla, infundibulum or fimbriae) is important, considering differences in cellular populations [68, 69].

Comprehensive and comparative molecular and functional analyses, including genomic and transcriptomic profiling, as well as drug response assays, should complement the histopathological evaluation to ensure the fidelity and translational relevance of patient-derived organoids [21, 22, 37].

## Conclusions

Despite the limited number of successfully generated patient-derived organoids, this study contributed to the evolving landscape of organoid models. The number of organoids established from HGSC, SBT/LGSC and FT remains below the ideal number for broader generalizability. This underscores the need for expanding our biobank with more cases, including treatment-naïve and post-treatment carcinoma specimens, to validate and extend these findings. Furthermore, this study underscored the need for a multidisciplinary collaboration in both clinical and research settings to establish these models as robust preclinical tools, allowing to expand our understanding of disease biology, supporting clinical decision-making, and, ultimately, improving cancer-related outcomes.

## Supplementary Information

Below is the link to the electronic supplementary material.

Supplementary Material 1

## Data Availability

No datasets were generated or analysed during the current study.

## Abbreviations

ACK: Ammonium-chloride-potassium
BSA: Bovine serum albumin
CK7: Cytokeratin 7
DAB: Diaminobenzidine
DAPI: 4′,6-diamidino-2-phenylindole
DMEM: Dulbecco’s Modified Eagle Medium
DPBS: Dulbecco’s phosphate-buffered saline
EGF: Epidermal growth factor
ER: Estrogen receptor
FGF: Fibroblast growth factor
FITC: Fluorescein isothiocyanate
FT: Fallopian tube
H&E: Hematoxylin and eosin
HGSC: High-grade serous carcinoma
IHC: Immunohistochemistry
LGSC: Low-grade serous carcinoma
PAX8: Paired box gene 8
PBS: Phosphate-buffered saline
PR: Progesterone receptor
ROCK: Rho-associated protein kinase
SBT: Serous borderline tumour
STIC: Serous tubal intraepithelial carcinoma
WT1: Wilms tumour 1
