# Supplementary material for: Patient-derived organoids as a model to study tubo-ovarian carcinoma: a pathologist’s perspective
Source: J Ovarian Res. 2025 Aug 20;18:191. doi: 10.1186/s13048-025-01766-4 (PMC12366147; doi:10.1186/s13048-025-01766-4)
Supplement: Supplementary file 1 — Supplementary Material 1 [file 13048_2025_1766_MOESM1_ESM.pdf]

SUPPLEMENTARY INFORMATION

Additional details on key materials and reagents used in the study

REAGENT /RESOURCE	MANUFACTURER	CATALOG NUMBER
**Antibodies and related**		
PAX-8 (clone MRQ-50)	Cell Marque (USA)	760-4618
WT1 (clone 6F-H2)	Roche (Switzerland)	760-4397
p53 (clone DO-7)	Roche (Switzerland)	800-2912
ERα (clone SP1)	Roche (Switzerland)	790-4324
PR (clone 1E2)	Roche (Switzerland)	790-2223
Ki-67 (clone 30-9)	Roche (Switzerland)	790-4286
OptiView DAB IHC Detection Kit	Roche (Switzerland)	760-700
ultraView Universal DAB Detection Kit	Roche (Switzerland)	760-500
ULTRA CC1 Antigen Retrieval Buffer	Roche (Switzerland)	950-224
Rabbit anti-Ki-67	Abcam (UK)	ab833
Mouse anti-EpCAM-FITC	Abcam (UK)	ab8666
Horse anti-mouse IgG secondary antibody, DyLight® 488	Vector Laboratories (USA)	DI-2488
Goat anti-rabbit IgG secondary antibody, DyLight® 594	Vector Laboratories (USA)	DI-1594
DAPI	Sigma (USA)	28718-90-3
µ-Slide 8 Well chambered coverslip	Ibidi (Germany)	80826
**Chemicals, peptides, and recombinant proteins**		
Advanced DMEM/F-12	ThermoFisher (USA)	12634-010
GlutaMAX™ Supplement	ThermoFisher (USA)	35050-061
HEPES	ThermoFisher (USA)	15630-080
Penicillin-Streptomycin	ThermoFisher (USA)	15070-063
Collagenase, Type II	ThermoFisher (USA)	17101-015
DPBS	Grisp (Portugal)	GTC13.0500
ACK Lysing Buffer	ThermoFisher (USA)	A1049201
Recombinant Human Noggin	Peprotech (USA)	120-10C
Recombinant Human R-Spondin1	Peprotech (USA)	120-38
B-27™ Supplement (50X), serum free	ThermoFisher (USA)	17504-044
N-Acetyl-L-Cysteine	Sigma (USA)	A9165
Nicotinamide	Sigma (USA)	N0636-100G
A83-01	Sigma (USA)	SML0788
Recombinant Human FGF-10	Peprotech (USA)	100-26
Animal-free Recombinant Human EGF	Peprotech (USA)	AF-100-15

Recombinant Human FGF-basic (FGF-2)	Peprotech (USA)	100-18B
Prostaglandin E2	Tocris (UK)	2296/10
SB 202190	Sigma (USA)	S7076
Y-27632 (Dihydrochloride)	StemCell Technologies (Canada)	72304
DNase I	Sigma (USA)	10104159001
Trypsin from bovine pancreas	Sigma (USA)	T9935
L Wnt-3A cells for production of Wnt-3A conditioned medium	ATCC (USA)	CRL-2647
Accutase® solution	Sigma (USA)	A6964
Formalin 10% solution neutral buffered	Bio-Optica (Italy)	05-01005Q
Paraformaldehyde	Sigma (USA)	P6148
Triton™ X-100	Sigma (USA)	T9284
Bovine Serum Albumin	Sigma (USA)	A2153
Tween® 20	Sigma (USA)	P1379
p-Phenylenediamine	Sigma (USA)	695106
Glycerol	Sigma (USA)	G6279
Matrigel® Basement Membrane Matrix, Phenol Red-free, LDEV-free	Corning (USA)	356237
HistoGel™	ThermoFisher (USA)	HG-4000-012
